# Active Teaching–Learning and Digital Technologies in Undergraduate and Postgraduate Dentistry Courses in Brazil

**DOI:** 10.1111/eje.70056

**Published:** 2025-10-22

**Authors:** Iara Vieira Ferreira, Larissa Stefhanne Damasceno deAmorim Póvoa, Beatriz Araújo de Souza, Daniele Sorgatto Faé, Francielle Silvestre Verner, Cleidiel Aparecido Araújo Lemos, Sibele Nascimento de Aquino

**Affiliations:** ^1^ Department of Dentistry Federal University of Juiz de Fora Governador Valadares Minas Gerais Brazil; ^2^ Postgraduate Program in Applied Health Sciences (PPGCAS) Federal University of Juiz de Fora Governador Valadares Minas Gerais Brazil

**Keywords:** active teaching–learning, dental curricula, postgraduate courses, undergraduate courses

## Abstract

**Introduction:**

Traditional methods versus active teaching–learning and new technologies have become increasingly important in health education, especially for training healthcare professionals for modern challenges. In dental education, active learning and digital technologies are recognised worldwide, but there is a lack of comprehensive analysis on their implementation in Brazilian undergraduate and postgraduate curricula.

**Materials and Methods:**

Data were collected from the official websites of undergraduate and postgraduate dentistry programs in Brazil. Descriptive and statistical analyses were performed using JASP software, with a significance level of *p* < 0.05. Chi‐square, Fisher's exact, and Mann–Whitney tests were applied to evaluate relationships between active methods, digital technologies, and course characteristics.

**Results:**

The study revealed low formal adoption of active learning methods and digital technologies in dental education. No significant association was found between active learning methods and general course characteristics. However, technology use was significantly associated with active learning methods in postgraduate and undergraduate courses.

**Conclusion:**

This study underscores the need for a more structured approach to preparing educators in both undergraduate and postgraduate dentistry programs to integrate active learning methods and digital technologies effectively. A clear framework for educator training is essential to meet modern educational demands and improve the quality of dental education in Brazil.

## 1 Introduction

In recent decades, numerous efforts have been made to enhance traditional teaching methods in education [1]. The growing interest in and discussion of active teaching‐learning approaches and new technologies is evident in the healthcare field, including dentistry [2], given the need to adequately prepare professionals for the complexities of contemporary practice [3, 4].

Active learning techniques, which involve engaging students in the process of acquiring knowledge, are considered one of the most promising educational strategies. Unlike traditional methods, active learning requires students to actively participate in activities, moving away from passive learning [5, 6]. Studies have shown that active learning is more effective than conventional methods [7, 8].

Various active teaching–learning techniques are described across educational levels, each with its own benefits and challenges [9]. In dentistry, methods such as problem‐based learning (PBL), team‐based learning [2], flipped classrooms [10], and case‐based approaches [11] have led to significant improvements in students' competencies and skills. However, implementing these methods poses challenges, requiring active engagement from both professors and students [12].

New technologies also play a crucial role in higher education, serving as either supplementary tools or primary methods in teaching [4]. Globally, digital technologies have been integrated into dental curricula to varying extents [13]. Tools such as intraoral optical scanning, 3D educational programs, web‐based learning, video demonstrations, mobile devices, dental simulators, and digital radiographs [14] offer pedagogical advancements, foster interactivity, facilitate resource management, and promote collaborative learning [4]. However, the rapid pace of technological advancement raises concerns about whether educational systems can keep up [3].

Despite these developments, little is known about the use of active methodologies and technologies in undergraduate and postgraduate dentistry programs in Brazil, aside from studies focusing on specific methods or tools. Therefore, this study aims to evaluate the integration of active methods and technologies in these curricula. The guiding question of this study is: ‘Are active teaching–learning methodologies and digital technologies implemented in graduate and undergraduate dentistry courses in Brazil, and how are they associated with these programs?’

## 2 Materials and Methods

This is a cross‐sectional study aimed at evaluating the official description of the use of active teaching‐learning methods and digital technologies in undergraduate and postgraduate dentistry courses in Brazil.

The *stricto sensu* postgraduate programs (master's and doctoral) were identified through the Sucupira Platform, the official database for postgraduate education in Brazil [15]. After identifying the courses, each program was accessed via its specific website. Data collection took place between January and August 2022, and all active postgraduate dentistry programs were included. The variables collected were the name of the institution, profile (public or private), state, region, program start date, number of available spots per year, graduate profile, number and type of courses (mandatory or optional), number of professors, use of digital technology in courses, and use of active teaching‐learning methods. These data were obtained from the course descriptions.

We also evaluated these variables for undergraduate dentistry programs in Brazil. The list of dental schools was verified on the official government website for education in Brazil [16], which lists all undergraduate programs in the country. Data were collected through access to the specific course websites, where institutional documents, curriculum matrices, and available information were reviewed. Extinct, endangered, and non‐initiated programs were excluded.

To determine the sample size for undergraduate programs, a calculation was performed using an effect size of 0.30, α error of 0.05, a power of 0.80, and a significance level of < 0.05, using G Power software, version 3.1.9.6. The institutions were then randomly selected. This calculation was performed to ensure sufficient statistical power to detect significant differences between groups (public vs. private institutions, and the presence of active teaching methods and digital technologies), thereby ensuring robust and representative results for dental programs in Brazil.

Institutional websites were systematically reviewed to assess the integration of digital technologies and active teaching–learning methodologies in dentistry programs. Data collection involved a structured analysis of course descriptions, curriculum matrices, and pedagogical project documents available on the official websites of each institution. The collected variables included institution name, profile (public or private), city, state, region, course start date, graduate profile, workload, and the explicit mention of digital technologies and active teaching–learning methodologies in the curriculum.

For this study, digital technologies were defined as tools that incorporate digital or electronic devices in teaching and learning. The specific technologies considered included 3D educational software, video demonstrations, mobile devices, and dental simulators, as identified in course descriptions and pedagogical documents. Similarly, active teaching‐learning methodologies were defined as instructional approaches that require direct student engagement beyond passive content reception. The methods analysed included PBL, flipped classrooms, and team‐based learning, as explicitly stated in official curricular materials.

The identification of these methodologies and technologies was based solely on the information available in institutional documents. As a limitation, it is acknowledged that this analysis may not fully reflect institutional practices, as not all methodologies and technologies in use may be formally documented or publicly disclosed online.

The data were analysed using JASP Team (2020) software, version 0.14.1 (University of Amsterdam), with *p* < 0.05 considered significant. The chi‐square test was used to compare categorical variables between groups when the expected frequencies were sufficiently large. For small sample sizes (*n* < 5) or when expected frequencies were low, Fisher's exact test was applied. For continuous variables, the Shapiro–Wilk test was first used to assess the normality of the data. If the data followed a normal distribution (*p* > 0.05), the student's t‐test was applied. If the data were not normally distributed (*p* < 0.05), the Mann–Whitney test was used. These statistical methods were chosen to ensure appropriate handling of the data distribution and to provide valid comparisons between groups.

## 3 Results

In 2022, 96 postgraduate courses in Dentistry in Brazil were identified on the Sucupira Platform. Among these, 17 were excluded due to insufficient information, duplicate data (for master's or doctoral degrees), or websites under maintenance. As a result, the analysis included 78 courses, with most (57.69%, *n* = 45) offered by public institutions. The Southeast region concentrated the highest number of programs (53%, *n* = 42), primarily in São Paulo (*n* = 27), followed by the South with 21.5% (*n* = 16). Fewer courses were identified in the Midwest (*n* = 5) and North (*n* = 3) regions.

Regarding the establishment of the courses, the most recent program was founded 3 years ago, whereas the oldest was established 63 years ago. Among the evaluated courses, 53 offered both master's and doctoral degrees, while 25 offered only a master's degree. On average, there were more vacancies available for master's programs (an average of 15 vacancies) than for doctoral programs (an average of 9 vacancies). The duration of student training ranged from 2 to 4 years, with an average of 18.65 professors' postgraduate programs. A diversity of training areas among professors was observed, contributing to variability within each course. The minimum number of compulsory subjects was 9, with an average of 12 for master's programs and 14 for doctoral programs.

Information on the use of active methodologies was available for 63 out of 78 postgraduate courses, with 9 (14.28%) reporting their implementation. The most commonly cited methods were blended learning and flipped classrooms. No significant associations were found between the adoption of active methodologies and factors such as institutional type (public or private, *p* = 1.00), years of existence of the course (*p* = 0.43), or the presence of a doctoral program (*p* = 1.00) (Table 1). Similarly, no significant differences were observed in the number of mandatory subjects in the master's program (*p* = 0.39), the number of mandatory doctoral subjects (*p* = 0.83), the number of professors (*p* = 0.66), the number of available spots in the master's program (*p* = 0.31), or the number of available spots in the doctoral program (*p* = 0.75).

**TABLE 1:** Postgraduate and undergraduate courses related or not to active teaching–learning and digital technologies.

Variables	Active teaching‐learning			Digital technologies		
Postgraduate	Active teaching‐learning (*n* = 63)			Digital technologies (*n* = 78)		
	Yes (*n* = 9)	No (*n* = 54)	*p* value	Yes (*n* = 9)	No (*n* = 69)	*p* value
Institution profile						
Public	5	28	1.00	5	40	1.00
Private	4	26		4	29	
Years of existence						
< 10 years	4	15	0.43	4	18	0.26
≥ 10 years	5	39		5	51	
Presence of PhD course						
Yes	6	36	1.00	6	47	1.00
No	3	18		3	22	

Undergraduate	Active teaching‐learning (*n* = 143)			Digital technologies (*n* = 108)		
	Yes (*n* = 38)	No (*n* = 105)	*p* value	Yes (*n* = 40)	No (*n* = 68)	*p* value
Institution profile						
Public	33	98	0.22	39	58	0.05
Private	5	7		1	10	
Years of existence						
< 10 years	19	40	0.20	22	33	0.51
≥ 10 years	19	65		18	55	
Number of semesters						
< 10	17	40	0.47	15	27	0.84
≥ 10	21	65		25	41	

Regarding the integration of technology in postgraduate courses, 9 out of 78 programs (11.53%) reported utilising applications, websites, spreadsheets, databases, and online activities. Notably, these were the same programs that also implemented active methodologies, with a significant association observed between the two (*p* < 0.001). Consistent with the findings for active teaching‐learning, no significant associations were found between technology integration and institutional type (public or private, *p* = 1.00), years of existence of the course (*p* = 0.26), or the presence of a doctoral program (*p* = 1.00) (Table 1). Similarly, no significant differences were noted for the number of mandatory master's subjects (*p* = 0.39), the number of mandatory doctoral subjects (*p* = 0.83), the number of professors (*p* = 0.65), the number of available spots in the master's program (*p* = 0.31), or the number of available spots in the doctoral program (*p* = 0.75).

Of the 486 undergraduate programs listed on the official site, 143 were evaluated. Among these, 131 (91.61%) were from private institutions, while 12 (8.39%) were from public institutions. The average minimum workload was 4345.9 h, ranging from 4000 h to a maximum of 6100 h, distributed across 8 (57%), 9 (3%), or 10 (60%) semesters. The median number of vacancies per year was 120. Among the evaluated courses, 26.57% (*n* = 38) utilised active methodologies. The use of active teaching–learning methods was not significantly associated with variables such as whether the institution was public or private (*p* = 0.22), the years of existence of the course (*p* = 0.20), or number of semesters (*p* = 0.47) (Table 1).

Information on the use of technology in undergraduate courses was available for 108 out of 143 programs, with 40 (37.03%) reporting the use of digital technologies in their classes. A wide range of technologies was mentioned, including virtual classes, digital platforms, simulation labs, interactive or multimedia classrooms, 3D technologies, e‐boards, cameras, websites, spreadsheets, databases, and virtual laboratories. Notably, the use of active methods was significantly associated with the use of technologies (*p* = 0.03). Although private institutions reported a higher use of technology (39/40, 97.5%), no significant association was found with institutional type (*p* = 0.05), years of course existence (*p* = 0.51), or number of semesters (*p* = 0.84) (Table 1).

## 4 Discussion

The current study represents, to the best of our knowledge, the first effort to explore the utilisation of active learning strategies and digital technologies in undergraduate and postgraduate dentistry courses in Brazil. The results showed that the formal insertion of active methods and digital technology is low in higher education in dentistry, especially in postgraduate courses. The results also showed that active methods were not associated with the general characteristics of the courses and that the use of technologies is associated with active teaching‐learning in undergraduates.

Co‐operative learning, PBL, case‐based learning, case studies, team projects, simulations and role‐playing, internships, peer tutoring, and challenging discussions, ability‐based education are common active learning strategies described [17]. A variety of benefits of active teaching‐learning include the development of student autonomy, changes in the traditional model, teamwork, integration between theory and practice, the development of a critical view of reality, and the favouring of formative assessment modalities [9, 18]. Despite the low numbers in official curricula observed by this study, it is important to highlight that some isolated experiences of the application of active methodologies in the teaching‐learning process in Brazil are performed in some isolated disciplines [18, 19, 20].

Recently, the Brazilian National Curriculum Guidelines determined the inclusion of active learning and technologies in the curricula to promote student learning in dentistry [15] and many institutions are now in the process of attending to the new guidelines. Thus, in the next few years, all undergraduates are expected to implement active methods, considering the mandatory nature of the new guidelines. It is interesting to note that these methodologies have been described as enhancing learning for many years [6], but even in the face of this data, a small portion of the undergraduates managed to insert. The lack of studies on active methodologies in dental education highlights that their implementation remains incipient, with challenges arising from the division between theory and practice in the traditional curriculum [21].

Our results indicate that active learning methods are more common in undergraduate dentistry programs than in postgraduate courses, possibly due to the lack of specific guidelines for their implementation. Despite the proven benefits of these methodologies, their adoption still faces challenges such as the need for prior preparation, limited time to cover content, lack of institutional resources, and resistance to change [22]. Moreover, the engagement of both professors and students is essential for successful implementation [12]. Although postgraduate dental education has expanded [23], there are still no clear pedagogical guidelines for these programs. Existing recommendations focus on incorporating innovation and technology [24], but they do not specify how these strategies should be applied in teaching, highlighting a gap in postgraduate education.

In this context, the necessity of change in the traditional system, with a guarantee of the training of professional educators, is fundamental [25]. It is important to highlight that most of the professors of higher education institutions had their training based on the so‐called ‘traditional’ model. Considering the low number of disciplines with active methods observed in our results, teacher training must be rethought, and the *stricto sensu* postgraduate course plays a fundamental role and must include not only technique but also the pedagogy and policy in which dentistry is inserted.

An interesting result of this study is that the use of digital technologies is significantly associated with active methods in undergraduates. In postgraduate courses, the same courses that describe the active methods in disciplines also cited digital technologies. This may reflect institutions that are already involved with teaching methods beyond the traditional, especially considering that technology in dentistry is widespread and offers added value to traditional teaching methods [26]. According to Lampropoulos and Kinshuk [27], integrating virtual reality and gamification in education makes learning more interactive, immersive, and effective. These safe and personalised environments enhance engagement, motivation, and academic performance while promoting cognitive and socio‐emotional development. For effective implementation, appropriate strategies, teacher training, and consideration of student characteristics are essential.

A systematic review showed the utilisation of web‐based learning techniques in the dental curriculum, comprising orthodontics, tooth anatomy, oral pathogens and immunology, dental radiology, oral surgery or implant dentistry, prosthetic dentistry, caries detection, growth and development, and the general use of web‐based learning tools. Video illustrations of clinical procedures with behaviour management in paediatric dentistry, intraoral suturing, or tooth preparation were described [14]. Some studies with technologies in Brazil have been reported and shown better performance of the students or improvement in teaching quality when technology was used [28, 29, 30, 31].

We observed a low number of postgraduate programs that cited the use of technologies in the disciplines. Interestingly, a qualitative study analysed the training and development of university professors of dentistry in *stricto sensu* postgraduate programs in Brazil, and the results showed that the training of university professors in dentistry is highly specialised and technologically focused and indicated the review of political‐pedagogic aspects of the educational sphere. The authors suggest that an evaluation of postgraduate *stricto sensu* programs is necessary [32].

It is important to highlight that today there are different generations in the population that include traditional and digital generations. Educators must realise that these differences are inevitable and concentrate on creating bridges and incorporating didactic experiences [33]. Active methods and digital technologies can bring these different generations closer and motivate students. However, it is necessary to promote a change in the mindset of the dental faculty and provide instructors with e‐learning and e‐learning training to enable the transfer of theoretical and practical knowledge [33]. Also, faculty need support to enable educators to use technology effectively for the benefit of their students [26].

Although this study is the first to explore the use of active methodologies and technologies in undergraduate and postgraduate dental programs in Brazil, several limitations should be highlighted. First, the data were exclusively obtained from institutional websites, which may not fully reflect actual teaching practices, as some institutions may adopt active learning strategies or digital technologies informally, without official online documentation. Furthermore, the study was conducted before the implementation of the new National Curriculum Guidelines, which now explicitly require the inclusion of active learning methodologies in undergraduate dental education. Therefore, with the implementation of these new guidelines, the adoption of active practices in dental education, both at the undergraduate and postgraduate levels, is expected to increase substantially in the near future. As a result, future investigations are needed to assess the impact of these changes on educational practices at higher education institutions in Dentistry in Brazil.

## 5 Conclusion

This study highlights that the adoption of active methodologies and digital technologies in Dentistry programs in Brazil remains limited, particularly in postgraduate education, where their integration is less frequent compared to undergraduate programs. The findings demonstrate a statistically significant association between the adoption of active methodologies and the integration of digital technologies in both undergraduate and postgraduate courses, suggesting that the successful implementation of active learning approaches can be enhanced by technological support. These results emphasise the need for a coordinated effort to modernise pedagogical practices in Dentistry.

Despite their well‐recognised effectiveness, the adoption of these approaches still faces challenges, such as the need for faculty training and, in the case of postgraduate education, the absence of specific guidelines to support their implementation. With the introduction of new National Curriculum Guidelines for undergraduate programs, significant progress is expected in the adoption of these practices. However, to ensure modernization also extends to postgraduate education, it is essential to establish clear guidelines and invest in faculty development at all levels. Therefore, continuously monitoring the impact of these changes and developing strategies to strengthen pedagogical innovation in higher education in Dentistry in Brazil is crucial.

## Ethics Statement

This study was performed in line with the principles of the Declaration of Helsinki.

## Conflicts of Interest

The authors declare no conflicts of interest.

## Data Availability

The data that support the findings of this study are available from the corresponding author upon reasonable request.
